# The effect of a roving nurse mentor on household coverage and quality of care provided by community health worker teams in South Africa: a longitudinal study with a before, after and 6 months post design

**DOI:** 10.1186/s12913-023-09093-4

**Published:** 2023-02-22

**Authors:** Jane Goudge, Olukemi Babalola, Hlologelo Malatji, Jonathan Levin, Margaret Thorogood, Frances Griffiths

**Affiliations:** 1 Centre for Health Policy, School of Public Health, Faculty of Health Sciences, University of the Witwatersrand, Johannesburg, South Africa; 2 School of Public Health, Faculty of Health Sciences, University of the Witwatersrand, Johannesburg, South Africa; 3 Warwick Medical School, Warwick University, Coventry, UK

**Keywords:** Community health workers, South Africa, Intervention, Supportive supervision

## Abstract

**Objective:**

Community health workers (CHW) are undertaking more complex tasks as part of the move towards universal health coverage in many low- and middle-income settings. They are expected to provide promotive and preventative care, make referrals to the local clinic, and follow up on non-attendees for a range of health conditions. CHW programmes can improve access to care for vulnerable communities, but many such programmes struggle due to inadequate supervision, low levels of CHW literacy, and the marginalized status of CHW in the health system. In this paper, we assess the effect of a roving nurse mentor on the coverage and quality of care of the CHW service in two vulnerable communities in South Africa.

**Participants:**

CHW, their supervisors, household members.

**Intervention:**

Roving professional nurse mentor to build skills of supervisors and CHW teams.

**Methods:**

Three household surveys to assess household coverage of the CHW service (baseline, end of the intervention, and 6 months after end of intervention); structured observations of CHW working in households to assess quality of care.

**Results:**

The intervention led to a sustained 50% increase in the number of households visited by a CHW in the last year. While the proportion of appropriate health messages given to household members by CHW remained constant at approximately 50%, CHW performed a greater range of more complex tasks. They were more likely to visit new households to assess health needs and register the household in the programme, to provide care to pregnant women, children and people who had withdrawn from care. CHW were more likely to discuss with clients the barriers they were facing in accessing care and take notes during a visit.

**Conclusion:**

A nurse mentor can have a significant effect both on the quantity and quality of CHW work, allowing them to achieve their potential despite their marginalised status in the health system and their limited prior educational achievement. Supportive supervision is important in enabling the benefit of having a health cadre embedded in marginalised communities to be realised.

**Supplementary Information:**

The online version contains supplementary material available at 10.1186/s12913-023-09093-4.

## Introduction

Community health workers (CHW) programmes have potential to improve access to care for vulnerable communities [1, 2], and they can be effective in improving health behaviours and outcomes [3, 4]. However, in many programmes the expected health benefits do not materialise as CHW have low levels of education, do not have adequate supervision, and are often marginalised as the lowest cadre, resulting in low motivation and poor performance [5]. With the call for universal health care coverage, and more recently the COVID pandemic, CHW are responsible for a greater range of promotive and preventative care, the skills required are broader and more complex, and the need for supervision is greater [6]. However, due to the shortage of health care workers in low- and middle-income settings, the number of nurses available to supervise CHW is limited.

South Africa has initiated a national CHW programme, known locally as the WBOT programme (ward-based outreach team), to strengthen primary health care [7]. The intention is to provide health promotion, prevention, screening and referral for a wide range of health conditions [8]. However, employing junior (enrolled) nurses as CHW supervisors has become the norm in many health districts, due to increasing salary costs and a shortage of professional nurses.

At the start of a 3-year intervention study, we studied CHW teams with different configurations of supervisors and locations. By comparing household coverage and quality of care, we concluded that both senior supervision, and proximity to, and support from, the clinic was important for CHW performance [9]. Teams with only junior supervisors, or based at health posts in the community, had less support from clinic staff, and CHW skills and credibility with clients was lower. Our intervention study, therefore, aimed to assess whether a roving professional nurse could mentor two CHW teams, building the capacity of the junior nurse supervisors, and the CHWs, and strengthen links with the clinic staff and local community structures. We aimed to determine whether any effects would be sustained once the roving nurse left. In this paper, we report on the effect of the intervention on the household coverage and the quality of the CHW service. The initial situational analysis [10, 11], and process evaluation accompanying this intervention study [12] are reported elsewhere.

## Background

In the South African programme, CHW teams consist of a nurse, six or more CHW, one health promoter and one environmental officer [7]. Each CHW team provides promotive and preventive services to households. Each CHW in theory cares for 250 families [9, 13].

CHWs, who had previously worked for NGOs, often providing home-based care during the height of the HIV epidemic, became the ward-based outreach teams in the new programme [9, 12]. While policy stated that the CHW had passed their final school matric exam to to work in the new programme, this requirement wasn’t always followed. Nation-wide standardized CHW training comprises three phases of learning, each with a written or practical examination, covering monitoring immunization, screening for malnutrition, adherence to long-term medication, gender-based violence, the identification of the need for antennal and post-natal care, making referrals to health and social services, and following up on patients who should visit the clinicI. In Sedibeng District, where the intervention took place, the CHWs also delivered chronic disease medications to the elderly and disabled patients [9, 12].

The supervisors are either professional or junior (enrolled) nurses. Professional nurses (PNs) in South Africa can diagnose, prescribe treatment and dispense medication. PN supervisors are trained in primary health care and community nursing. Junior nurses (ENs) complete a 2-year nursing course and are qualified to provide nursing care under supervision. While official policy documents acknowledge the importance of supportive supervision for CHW, there is little guidance as to how this should be operationalized, given the limited resources [14]. Supportive supervision has been defined as having three key functions: management (ensuring compliance with organizational standards, engagement with other stakeholders outside of the immediate team); education or development (seeks to improve knowledge and skills); and support (seeks to ensure morale, motivation and job satisfaction) [14, 15]. It is this definition that we used in this study.

## Methods

### Study design

**Fig. 1:**
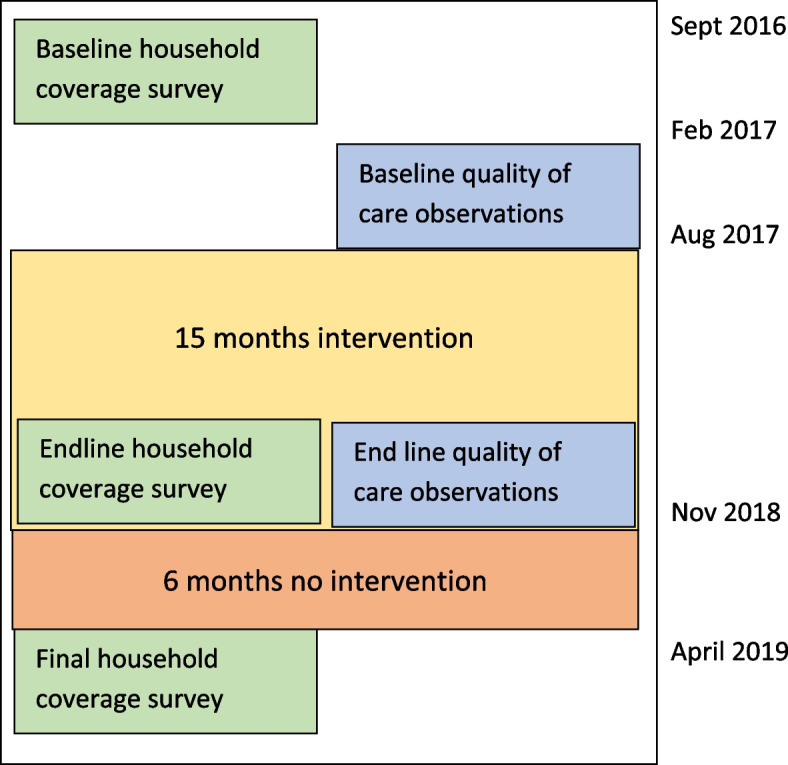
Phases of intervention and data collection

We assessed the effect of the intervention using a before, after, and 6 months post-intervention study design (see Fig. 1). Initially we conducted a situation analysis in September 2016 – February 2017 to study 6 CHW teams with different supervision and location configurations [9]; this situational analysis included a survey to assess household coverage which was used as the baseline in the intervention study. The six teams included two facility-based teams supervised by a professional nurse and a junior nurse, two health-post teams supervised by a professional nurse and a junior nurse, and two facility-based teams supervised by a junior nurse only.

The intervention was implemented from August 2017 to November 2018. In total, we conducted a second household survey to assess household coverage at end of the intervention (endline), and the third 6 months after intervention had ended to assess whether any benefits from the intervention were sustained (final) (Fig. 1). We observed CHW visits to households at baseline and endline to assess the quality of care provided. We did not assess the quality of care 6 months after the intervention, as initial analysis showed no change between baseline and endline in our primary outcome variable (the proportion of appropriate messages given by CHW as observed by a fieldworker).

### Study site

In Sedibeng District, although relatively well off by South African standards, nearly a quarter of the residents have insufficient food to eat [9, 12]. Disadvantaged communities with inadequate housing or food and high levels of illness have limited access to health services, and also, transport networks, water and electricity. In the two sites, which are located 30 km fromthe nearest town, residents’ housing was government provided housing (small brick houses) or shacks made of plastic and re-used corrugated iron. As in rural areas in South Africa, the majority of the inhabitants were without work and relied on government grants [9, 12].

#### The CHW teams

Both team leaders had completed a 2-year nursing qualification and had a two of years’ work experience although not with CHWs before [9, 12]. One of the supervisors (Team 1) was from another province and sometimes appeared uncomfortable overseeing the CHWs; CHWs were local women and older than her. The supervisor in Team 2 was from the same local area; she was also more assertive and the CHWs in her team were younger (Table 3) [9, 12].

The CHWs’ had been working for between 3 – 17 years (Table 1). Three-quarters of the CHWs in Team 1 and nearly a fifth of CHWs (of 34) in Team 2 had not finished their schooling. Only 2 CHWs in Team 1, and 1 CHW in Team 2 had passed Level 1 CHW training. None of the CHWs had passed the Levels 2 or 3 CHW training [9, 12].

**Table 1:** CHW team characteristics

**Supervisor**	Team 1	Team 2
No. of enrolled nurses	1	1
Age (years)	36	31
Years as nurse	5	2
Years in programme	0.3	0.3
**CHW**		
No. of CHWs per team	14	20
Mean age in years (range)	42 (23–58)	33 (23–54)
Mean years as CHW (range)	10 (3–9)	6 (5–17)
Proportion of CHWs who have finished high school education	25%	33%
No. of CHWs who have passed phase 1 training	2	1
No. of CHWs who have passed Phase 2 training	0	0

### The intervention

Given the findings of our situation analysis and the national shortage of experienced professional nurses, in discussion with district and provincial stakeholders, we agreed our intervention would be to employ an experienced professional nurse (‘a nurse mentor’) on a full time basis, who had experience in community nursing and adult training. She would move between the two facility-based teams with junior nurse supervisors over a period of 15 months. The intention was a nurse-mentor could then move on to support other CHW teams and their supervisors in the district, if such a person was employed by the health services. (While the nurse-mentor for the study was employed by Wits University, and reported to the study team, District managers were part of the recruitment panel, and there was full consultation and agreement about her role before she started.)

The first aim of intervention was to mentor the team as a whole. This included increasing the clinical knowledge and skills in client engagement of both the supervisors and the CHW. It also included providing, and role modelling, supportive supervision such that the supervisors could improve their supervision skills, and that the CHW could understand the benefits of such supervision.

The second aim was to strengthen relationships between the CHW team and clinic staff, and the third, to strengthen relationships with community organisations and political structures. The appointed nurse mentor had a 4-year nursing degree and 15 years’ experience in nursing of which 6 years was in supervisory roles in other CHW programmes. The nurse mentor first observed some well performing CHW teams in the same district, and then spent 2 weeks observing and learning about her two assigned sites, gradually moving into a mentorship role, dividing her time between the two sites.

The nurse mentor provided both the EN supervisors and CHWs with training on a range of clinical topics (BP and blood glucose measurement, malnutrition, appropriate ANC and PNC care, when to refer patients) (*education function*). She facilitated role plays where ENs and CHW could practice engaging with household members, and then accompanied ENs and CHW on household visits, supporting them as they practiced their new skills *(support function)*. She supported and supervised the two EN supervisors, demonstrating how to encourage the CHW to practice new skills, how to provide supportive supervision during household visits with the CHW and to consolidate new learning in debriefing sessions at the end of the day (*education and support function*).

She established new working practices (e.g., a book to record which patients required medication delivery and when) and held the CHW accountable for any absenteeism (*management function*). When the clinic photocopier wasn’t working the nurse mentor provided forms, so that work could continue. She held meetings with the facility manager, staff members (data clerks and the pharmacy assistant) and engaged other staff to discuss new work practices she had established (*management function*), and what help the CHW required from the staff (*support function*). She encouraged the CHW when their efforts were dismissed by facility staff, and when they doubted their own ability to pass the necessary tests (*support function*). She supported the supervisors so that they could take on these management and support functions once she left.

CHW supervisors are responsible for community engagement. The nurse mentor, accompanied by the supervisors, held meetings with local political leaders where possible (e.g., ward counsellor) and relevant local organisations (e.g., local NGO, traditional healers, clinic committee, school principals, creche managers). A public community meeting provided the opportunity to meet several stakeholder organisations to explain the work of the CHWs, and to discuss how the CHW might work with the various stakeholders. The leaders within both communities were not responsive to the nurse mentors’ efforts.

The nurse mentor initially spent 2 months with the first team, and once the new practices were sufficiently well established, she then moved to the second team, again spending 2 months. She then rotated between the two facilities, allowing the supervisors to take charge in her absence and demonstrate their capability to manage the teams on their own. (More detail how the intervention evolved over the study period, and mechanisms by which it worked, is provided in the paper reporting process evaluation.)

### Data collection

Table 2 below sets out the data collection methods, number of participants and data that were collected. Data collectors were recruited and trained on community orientated healthcare, data collection techniques and research ethics. The data collection took place from October 2016 –September 2019.

**Table 2:** Household surveys to assess coverage, and visit observation to assess quality of care

Surveys		Number		Data collected
		Site A / Team 1	Site B / Team 2	
Household coverage survey	Household interviews			• Socio-economic status of household • Demographic profile; • Need for, and access to care • Whether a CHW had visited in the past month/year (coverage);
	Baseline	206	209	
	Endline	488	445	
	Final	590	618	
Quality of care observations	Observed household visits			• Working equipment • Prior engagement with household by CHW • The health conditions identified by CHW, messages given by CHW and actions planed • Aspects of communication during visit (e.g. whether consultation took place outside or inside house; whether the households demonstrated a negative attitude to the CHW; whether the visit was disrupted or not)
	Baseline	148 (12 CHW)	148 (12 CHW)	
	Endline	110 (16 CHW)	106 (6 CHW)	

#### Household survey to assess coverage by CHW programme

We used stratified sampling to select 220 households per site for the baseline, 475 for the endline, and 600 for the final survey. (The sample size was increased in subsequent surveys to provide enough power to detect a 10% change.) Stratification was by area, using an estimate of the population in that area, as each community tends to have a similar housing type, reflecting socio-economic status (e.g., informal settlements compared to community with formal brick houses.) Data collectors used a random walk and a specified skip pattern in each designated area to select households. The household was approached and the member who knew most about the health of other members was invited to participate in the survey. Their responses were recorded on an electronic device. This allowed any irregularities to be identified and resolved as the survey progressed. We collected data on socio-economic status, the need for care (number and types of health conditions), and coverage (whether a household had been visited in the past year or in the past month). Descriptive statistics were generated for all variables of interest and compared across sites and time.

A logistic regression analysis was conducted to identify the factors associated with the change in the likelihood of a household being visited in the last month. We included the following as categorical variables: site (site A vs site B), dwelling type (House vs Informal), distance from the facility (30 vs 30-45 vs >45 minutes), whether the household has a person over 60 (yes vs no), a child under 5 (yes vs no), hypertension (yes vs no), diabetes (yes vs no), HIV (yes vs no), cough more than 2 weeks (yes vs no), pregnancy (yes vs no), and time was entered as a dummy variable for the different intervention periods (Time 1 [baseline], 2 [endline], and 3 [sustainability]). We first conducted an unadjusted analysis to determine whether any of the variables were significantly associated at the 20% level (*p* ≤ 0.2) with each explanatory variable included in a model with the outcome (visited or not), one after the other in individual unadjusted models. In the adjusted model, all independent predictors that were significant at 20% level (*p* ≤ 0.2) in the unadjusted were included initially, and those which are not significant (now using the 5% level) were removed one by one, with the least significant being removed first. When building the adjusted model, regardless of whether site, dwelling type and distance were significant or not, they were retained in the model.

#### Observations of household visits to assess quality of care

We developed an observation tool (QoCAT) to assess the quality of care delivered by CHWs before and after the intervention [16]. We used qualitative data from the situation analysis to develop the QoCAT, and an associated fieldwork manual. (The tool and the manual is provided as supplementary files in [16]). The sections of the tool followed the flow of the working day for CHW: before setting out, then for each household visit - just before entry, during household visit and, after leaving the household. The tool was used to record the purpose of the visit, basic demographic information about each client, and the health conditions that were reported. The observer selected from a predefined list for each condition reported which health messages were provided by the CHW. For example, for hypertension there were four expected messages and actions: (a) asking and advising about food/exercise, (b) asking about medication adherence/side-effects, (c) measuring blood pressure and (d) checking access to medication supplies. If a householder had a condition such as hypertension, the community health worker was scored on the number of messages or actions delivered. Based on the number of reported conditions, we calculated the proportion of expected messages that were given. (The items were equally weighted.)

The tool was also used to record information about the communication during the visit: did the conversation take place on the doorstep/outside or in the house; was the visit disrupted (e.g. by a child); did the client seem to have a negative attitude towards the CHW; did the client discuss barriers to seeking care; did the CHW take notes of the visit. The fieldworker also asked CHW which equipment items she had in her bag; this was also recorded on the tool.

Most items require a categorical response (e.g., present/absent). We used role plays with fieldworkers to refine the tool. The tool was piloted by the fieldworkers at a site different from our study sites. We observed CHWs for a three-day period (to reduce the Hawthorne effect). We selected which CHW to observe by placing the names of all the CHW present on day 1 of each 3-day period in a box and randomly drawing out a name. We observed a total of 296 household visits at baseline and 216 at endline.

We conducted double data entry using REDCap software, and differences were discussed and resolved. The proportion of appropriate health messages actually given by the CHW was used to calculate a household message score. The communication score for each household visit was the proportion of items assessing communication for which a positive outcome was recorded. All statistical analysis was carried out using Stata version 14. Descriptive statistics for continuous variables were summarized using both the mean and standard deviation and median and interquartile range (IQR). Categorical variables were described using frequency tables.

### Ethics

The study was given ethical clearance by the University of the Witwatersrand Human Research Ethics Committee (Medical) (M160354). Respondents gave written informed consent. Householders gave permission for the fieldworker to observe the CHW in the household. To ensure participants anonymity, unique codes were allocated to participants.

### Patient and public involvement

CHW clients and the public were not involved in the design, conduct, reporting or dissemination plans; however, facility, district and provincial managers were involved. We have conducted feedback sessions with CHW teams, facility, district and provincial managers.

## Findings

### Coverage from the household survey

#### Description of households

The three household surveys in the two sites had on average a response rate of 95.8%. In Site A the proportion of informal dwellings fell from 24 to 9% due to a government housing programme. In Site B approximately half of households were informal dwellings throughout the study. Access to the internet rose steadily in both sites from 25-30% to 50-70% households. The proportion of households with just one person rose from 15 to 24% across the two sites. The proportion of households who were either less 30 mins, 30-45 mins or more than 45 mins walk to the facility remained similar across the three surveys in both sites, suggesting that our comparison of coverage in the three time periods would not be influenced by CHW having to walk differing distances to the surveyed households.

#### Coverage

Household coverage by the CHW rose considerably between baseline and endline (Table 3). The proportion of households visited by a CHW in the last year in Site A rose from 19.9 to 31.4% of households, and then fell by 1%. In Site B, coverage doubled from 19.6% of households to 40.3% in but then fell to 30.0%.

**Table 3:** Coverage and health conditions recorded in baseline, endline and final surveys

Site	Site A / Team 1						Site B / Team 2					
Survey	Baseline		Endline		Final		Baseline		Endline		Final	
	n	%	n	%	n	%	n	%	n	%	n	%
**All households:**	206		504		621		209		501		621	
Ever visited by a CHW	47	22.8	188	37.3	226	36.4	43	20.6	222	44.3	208	33.5
Visited in last month	12	5.8	103	20.4	122	20.0	26	12.4	114	22.8	116	18.7
Visited in last year	41	19.9	158	31.4	187	30.1	41	19.6	202	40.3	186	30.0
**Proportion of households reporting a health condition, who were visited:**												
Ever visited by a CHW	35/139	25.2	144/339	42.5	171/412	41.5	33/124	26.6	162/314	51.6	168/410	41.0
Visited in last month	7/139	5.0	82/339	24.2	101/412	24.5	22/124	17.7	79 /314	25.2	94/410	22.9
Visited in last year	30/139	21.6	122/339	36.0	144/412	34.9	31/124	25.0	148/314	47.1	151/410	36.8
**Proportion of households with a member reporting the following conditions, who were visited in the last year:**												
Hypertension	14/62	22.6	81/187	43.3	67/162	41.4	14/42	33.3	75/154	48.7	73/200	36.5
Diabetes	4/24	16.7	22/56	39.3	20/34	58.8	6/17	35.3	18/43	41.9	13/24	54.2
HIV	13/47	27.7	51/126	40.5	40/116	34.5	5/29	17.2	43/91	47.3	43/126	34.1
≥ 60 years	2/12	16.7	49/101	48.5	49/104	47.1	5/20	25.0	42/84	50.0	32/78	41.0

The denominates vary due to varying number of people for whom the variable of interest was relevant

While Team 2 (Site B) had a greater increase in coverage during the intervention, the CHW weren’t able to sustain that increase. Team 2 had a more competent supervisor who applied for training to become professional nurse at the end of the intervention, however, her application was not successful and as a result she lost considerable motivation. Six months after the intervention both sites had increased their coverage by just over 50% from baseline.

The results of the fitted logistic regression indicated that a household was more than twice as likely to have been visited in the past month at endline (aOR 2.65; *p*value<0.001) and 6 months post endline (aOR 2.39; *p* value<0.001), compared to prior to the intervention. The results showed that households with a member who was either elderly (over 60) (aOR 2.09; *p* value<0.001), under 5 (aOR 1.35; *p* value 0.017) or who had hypertension (aOR1.59; *p* value<0.001), HIV (aOR 1.43; *p* value 0.049) or TB (aOR 2.28; *p* value 0.009) were more likely to be visited in the last month. Across the three time points, the socio-economic status of the household (as indicated by whether the dwelling was a formal brick building or an informal shack) was not significantly associated with a visit in the last month, nor was the walking distance between the clinic and the house, suggesting CHW didn’t prioritise households based on distance from the clinic or socio-economic status.

### Quality of care

#### Equipment

At both baseline and endline, over 85% of CHW bag checks revealed a working BP machine. Over a third had glucose strips and a working glucometer at baseline but at the end of the intervention this had dropped to zero. The glucometers and strips had been issued along with the BP machines, but the glucose test strips were never replenished. CHW in Team 1 didn’t carry the bathroom scales issued for weighing children and adults to household visits as they were heavy.

#### Type of visits and clients

At baseline, three-quarters and two-thirds of CHW visits (77 and 62%) were medication delivery in site 1 and 2 respectively (Table 4). At endline this dropped to one third of visits (36 and 37%), with an increase in all the other types of visits, particularly household registration. Registration requires the CHW to approach an often-unknown household, assess health needs and recommend appropriate action, a more complex task than delivery of prescribed medication. The change in the type of visit also changed the frequency of visits, with fewer repeat visits to the same household. In both sites the CHW engaged with a greater variety of age groups (including children and pregnant women) and fewer people over 60/retired/elderly (to whom medication delivery service was directed).

**Table 4:** Description of visits observed using the QoCAT tool

	Site A / Team 1				Site B / Team 2			
	Baseline		Endline		Baseline		Endline	
**Number of household visits observed**	148 (100.0)		110 (100.0)		148 (100.0)		106 (100.0)	
**When previously visit by CHW**	n	%	n	%	n	%	n	%
Never	13	8.8	42	38.2	12	8.1	27	25.5
In the last week	99	66.9	37	33.6	111	75.0	28	26.4
In the last month	28	18.9	19	17.3	23	15.5	40	37.7
In the last 3 months	7	4.7	8	7.3	2	1.4	9	8.5
In the last year	1	0.7	3	2.7	0	0.0	2	1.9
More than a year ago	0	0.0	1	0.9	0	0.0	0	0.0
**Current visit plan**								
Household registration	20	13.5	52	47.3	14	9.5	25	22.1
Medication delivery for patients over 60 years	114	77.0	40	36.4	93	62.9	42	37.2
Child/baby check	7	4.7	11	10.0	13	8.8	22	19.5
Pregnant woman check	1	0.7	4	3.6	1	0.7	4	3.5
Defaulter tracing for clinic	2	1.4	3	2.7	3	2.0	7	6.2
Other checks including elderly	4	2.8	15	13.6	24	16.3	13	11.5
**Interaction with householder**								
Took place at yard gate or doorstep	17	11.5	3	2.7	31	20.9	20	18.9
Difficult as communication difficulty	1	0.7	3	2.7	9	6.1	4	3.8
Negative towards CHW	5	3.4	4	3.7	6	4.1	6	5.6
Disrupted e.g., TV, crying child	0	0.0	10	9.4	0	0.0	20	18.9
Barriers to care e.g., transport, attitude of nurses	0	0	10	9.4	0	0	7	6.6
Reveals other problems	0	0.0	14	13.2	0	0.0	9	8.4
No notes taken by CHW	49	33.1	19	17.3	67	45.3	23	21.7

Fewer visits took place on the doorstep, or in the yard, rather than in the house. A greater number of householders discussed the barriers they faced in accessing care suggesting fuller conversations were happening, and that the CHW were gaining the confidence of householders (Table 4). In both sites, by the end of the intervention, there were more visits where CHW took notes about the visit. The number of clients who were given a referral letter increased in both sites, perhaps due to the improved availability of the forms during the intervention, and the CHW were more likely to plan to revisit the household at the endline.

While the proportion of appropriate messages given didn’t change (it remained at just over 50% in both sites), (perhaps because only so much advice can be taken on board by households), there was a shift in the focus of the messages given. CHW tended to not only provide advice about being adherent to medication, but to check on whether the client made a clinic visit on time, for example, to collect a repeat prescription (Table 5). CHW were more likely discuss a child’s Road to Health card (a parent held record of child health), feeding, and attending the clinic for immunisations. CHW was also more likely to ask questions about possible new health needs, such as family planning needs, whether anybody had a persistent cough, or HIV status.

**Table 5:** Proportion of messages provided by condition and message

	SITE A / Team 1				SITE B / Team 2			
	Baseline		Endline		Baseline		Endline	
**Number of HH**	**148**		**110**		**148**		**106**	
**Number of clients**	**189**		**169**		**234**		**148**	
	n	%	n	%	n	%	n	%
**Hypertension**	**127**		**53**		**56**		**38**	
Lifestyle advice	62	48.8	29	54.7	27	48.2	23	60.5
Taking medication	107	84.3	32	60.4	52	92.9	26	68.4
Accessing medication	37	29.1	33	62.3	20	35.7	25	65.8
Measuring BP	95	74.8	31	58.5	34	60.7	33	86.8
**Diabetes**	**31**		**11**		**28**		**6**	
Lifestyle advice	17	54.8	5	45.5	16	57.1	2	33.3
Taking medication	24	77.4	8	72.7	27	96.4	5	83.3
Accessing medication	21	67.7	7	63.6	16	57.1	6	100.0
Measuring BP	11	35.5	3	27.3	0	0.0	2	33.3
Measuring blood glucose	3	9.7	1	9.1	1	3.6	0	0.0
Foot care	11	35.5	3	27.3	12	42.9	2	33.3
**HIV**	**16**		**14**		**41**		**33**	
Lifestyle advice	7	43.8	3	21.4	15	36.6	13	39.4
Taking medication	15	93.8	10	71.4	34	82.9	26	78.8
Accessing medication	14	87.5	6	42.9	32	78.0	28	84.8
**Persistent cough**	**22**		**3**		**8**		**2**	
Screening questions for TB	20	90.9	3	100.0	8	100.0	2	100.0
**Child 6 months to 5 years**	**10**		**26**		**25**		**24**	
RTH card	2	20.0	19	73.1	10	40.0	21	87.5
Feeding/diet/grant	5	50.0	8	30.8	2	8.0	7	29.2
Advice on attending clinic	6	60.0	20	76.9	11	44.0	16	66.7
HIV status	2	20.0	5	19.2	2	8.0	3	12.5
Looking at/measuring child	9	90.0	14	53.8	21	84.0	15	62.5
**Routine Check**	**138**		**154**		**190**		**131**	
^a^Family planning	10 /28	35.7	36 /61	59.0	22/75	29.3	31/60	51.7
HIV status	3 /138	2.2	55 /154	35.7	15/ 190	7.9	56/131	42.7
^a^Pap smear	2/100	2.0	25/94	26.6	19/124	15.3	14/77	18.2
Cough	33/138	23.9	66 /154	42.9	54/56	96.4	32/131	24.4
Social grants	11 /138	8.0	77 /154	50.0	23 / 190	12.1	36/131	27.5
Check BP	6 /138	4.3	36 /154	23.4	18 / 190	9.5	38/131	29.0
Are you sick?	107 /138	77.5	102 /154	66.2	164 / 190	86.3	61/131	46.6
^a^Any one pregnant	2/28	7.1	31/61	50.8	4/75	5.3	21/60	35.0
^a^Birth in last 6 weeks	0/28	0.0	11/61	18.0	0/75	0.0	8/60	13.3
^a^Is this a child under 5	2/3	66.7	19/28	67.9	2/15	0.1	20/27	74.1
Does this person take daily medication	6 /138	4.3	96 /154	62.3	24/ 190	0.1	61/131	46.6
Check glucose	17 /138	12.3	4/154	2.6	0 / 190	0.0	2/131	1.5
^a^Prostate cancer	0/34	0.0	0/31	0.0	1/47	2.1	0/26	0.0
check on known elderly person	8 /138	5.8	36 /154	23.4	6 / 190	3.2	15/131	11.5

^a^Denominator varies due to smaller number of family members to whom this question was relevant

## Discussion

The intervention of the nurse mentor led to a sustained 50% increase in household coverage. CHW were more likely to visit new households, to provide care to a greater range of people and performed a greater range of more complex tasks. This evidence suggests that a nurse mentor providing training and supportive supervision can have a significant effect both on the quantity and quality of CHW’s work.

There is small and growing number of intervention studies examining the impact of supportive supervision in CHW programmes [17–19]. Often the content and activities of the supportive supervision is not fully described, rather authors tend to state the frequency (often once or twice a month) and length (often 2-3 hours) [19, 20]. One exception is Kok et al. (2018) who define supportive supervision as being concerned with: a) the supervisees’ welfare; b) educating supervisees; b) ensuring performance standards are met [17].

Several studies have focused on better communication between CHW and their local supervisor, and better access to supplies. For example, improved supervision through use of a mobile phone app through which data is shared on diagnosis, treatment, referrals [21], or through more frequent supervisor visits [19], or the use of sms to request a supervisor visit [22]. Of these three studies, only the latter showed any impact on CHW performance. These studies did not consider the content of the supervisory exchange in terms of whether it was supportive (or not).

Two studies focused on providing training for supervisors [17, 18]. The qualitative data showed that the sense of joint responsibility, teamwork and cross-learning were valued [17, 18] An unexpected consequence of the supportive supervision was an increase the CHWs’ participation, and confidence to voice any concerns at the intervention facilities [18]. In one study supportive supervision led to a non-threatening and empowering environment in which the supervisor and the CHW volunteer could learn and overcome obstacles together; the intervention villages had better outcomes than the control sites [20].

When assessing the impact of supportive supervision, the choice of outcome measure is complex. Where the aim is to improve skills in a specific activity (e.g. provision of iCCM services [21], or encouraging treatment seeking or use of bed nets [19]) and the outcome measure is directly related to that activity, studies often identified an improvement in outcomes; however, where the outcomes were the rather more distal measures such as job satisfaction or organizational commitment, available studies did not show an improvement [17, 18]. We wished to assess abilities in wide range of activities, and so decided not to focus on specific tasks. The quantity of health messages given may not have been sufficiently sensitive to changes in the quality of care, but the recording of the different messages given, and topics of conversation in a household visit, led to nuanced understanding of the CHWs activities.

### Policy and practice implications

In the South African context, we anticipate that one nurse-mentor could support with 12-15 teams, although this depends on how much support on-going support the local supervisors need, and the distances between the location of the teams and where the mentor is based. In other supervisory systems, ie facility-based, peer, group or community supervision [23], it is difficult to anticipate the implications of our findings. We would recommend that those interested consult the paper in which we report the accompanying process evaluation to obtain a fuller understanding the processes that led to change [12], as well as the other accompanying papers [10, 11] and to generate ideas as to how relevant similar but locally relevant process might be initiated.

### Strengths and limitations

We provide a detailed description of our intervention and assessment of quality of care provided by CHWs. While our study lacked a control group, the post-intervention assessment was useful in assessing whether the effect was sustained. The lack of change in the quality of care as assessed by the QoCAT tool may indicate a low level of validity, but as discussed above there is no gold standard for how to assess CHW quality of care. (Using standardised or mystery patients is not possible as the care takes place in people’s homes.) There were several instances of strike actions that occurred during the study due to the quest by CHWs for improved conditions of services (salary and permanent employee of government) from the government. However, the number of working days lost was approximately 15 during the intervention period, which we did not consider to be sufficient to have an impact on the study results. Due to the long duration of the study, inevitably, there was turnover among the fieldworker team. However, extensive training and piloting, were conducted with each team before any field work.

## Conclusion

Our results suggest that a roving nurse mentor providing supportive supervision focused on all three of the key functions of supervision, can lead to a substantial improvement in CHW performance. CHW, because of their marginalised status in the health system, need supportive supervision to ensure the development of appropriate work systems, to improve their skills, enabling greater credibility among colleagues and clients.

## Supplementary Information

**Additional file 1: Supplementary Table 1**. Description of dwellings and households. **Supplementary Table 2.** Description of household respondents. **Supplementary Table 3.** Factors associated with being visited (unadjusted and adjusted regression analysis): both sites combined.

## Data Availability

The datasets used and/or analysed during the current study available from the corresponding author on reasonable request.

## Abbreviations

CHW: Community Health Worker
DoH: Department of Health
QoCAT: Quality of care assessment tool
