# Supplementary material for: The effect of a roving nurse mentor on household coverage and quality of care provided by community health worker teams in South Africa: a longitudinal study with a before, after and 6 months post design
Source: BMC Health Serv Res. 2023 Feb 22;23:186. doi: 10.1186/s12913-023-09093-4 (PMC9948528; doi:10.1186/s12913-023-09093-4)
Supplement: Supplementary file 1 — Additional file 1: Supplementary Table 1. Description of dwellings and households. Supplementary Table 2. Description of household respondents. Supplementary Table 3. Factors associated with being visited (unadjusted and adjusted regression analysis): both sites combined. [file 12913_2023_9093_MOESM1_ESM.docx]

**Supplementary Table 1: Description of dwellings and households**

| **Site** | **Site A / Team 1** | | | | | | **Site B / Team 2USI** | | | | | |
| --- | --- | --- | --- | --- | --- | --- | --- | --- | --- | --- | --- | --- |
| **Survey** | **BASELINE** | | **ENDLINE** | | **SUSTAINABILITY** | | **BASELINE** | | **ENDLINE** | | **SUSTAINABILITY** | |
|  | **n** | **%** | **n** | **%** |  | |  |  |  |  |  | |
|  | **209** | **100.0** | **504** | **100.0** | **621** | **100.0** | **206** | **100.0** | **504** | **100.0** | **621** | **100.0** |
| Informal dwelling | 90 | 43.1 | 243/500 | 48.6 | 310 | 49.9 | *47/198 | 23.6 | 69/500 | 13.8 | 58 | 9.3 |
| Access to piped water | 162 | 77.5 | 479 | 95.6 | 580 | 93.4 | 163 | 79.1 | 487 | 96.6 | 609 | 98.1 |
| Access to toilet | 194 | 92.8 | 478 | 95.4 | 578 | 93.1 | 167 | 81.1 | 488 | 96.8 | 607 | 97.7 |
| Access to internet | 53/209 | 25.4 | 229 | 45.7 | 352 | 56.7 | 67 | 32.5 | 287 | 56.9 | 457 | 73.6 |
|  |  |  |  |  |  |  |  |  |  |  |  |  |
| Size of household |  |  |  |  |  |  |  |  |  |  |  |  |
| I person | 30 | 14.4 | 111 | 22.2 | 150 | 24.2 | 30 | 14.6 | 74 | 14.7 | 134 | 21.6 |
| 2-4 persons | 135 | 64.6 | 270 | 58.9 | 359 | 57.8 | 120 | 58.2 | 293 | 58.1 | 342 | 55.1 |
| 5-10 persons | 41 | 19.6 | 117 | 23.3 | 111 | 17.9 | 55 | 26.7 | 133 | 26.4 | 138 | 22.2 |
| 11 or more | 3 | 1.4 | 3 | 0.6 | 1 | 0.2 | 1 | 0.5 | 4 | 0.8 | 7 | 1.1 |

*8 records missing so denominator is 198 instead of 206

**Supplementary Table 2: Description of household respondents**

| **Site** | | **VIS** | | | | | | **USI** | | | | | |
| --- | --- | --- | --- | --- | --- | --- | --- | --- | --- | --- | --- | --- | --- |
| **Survey** | | **BASELINE** | | **ENDLINE** | | **SUSTAINABILITY** | | **BASELINE** | | **ENDLINE** | | **SUSTAINABILITY** | |
|  | | **n** | **%** | **n** | **%** |  | |  |  |  |  |  | |
| Total number of household members | | 679 | 100.0 | 1770 | 100.0 | 1857 | 100.0 | 722 | 100.0 | 1898 | 100.0 | 2043 | 100.0 |
| Mean number of persons per household (SD) | | 3.25  (range 1-11) | 1.86 | 3.26 | 2.06 | 2.98  (range 1-14) | 1.94 | 3.51  (range 1-11) | 1.94 | 3.68 | 3.24 | 3.29  (range 1-14) | 2.13 |
|  | |  |  |  |  |  |  |  |  |  |  |  |  |
| Gender | |  |  |  |  |  |  |  |  |  |  |  |  |
| Male | | 329 | 48.5 | 882 | 49.8 | 866 | 46.6 | 336 | 46.5 | 907 | 47.8 | 972 | 47.6 |
| Female | | 350 | 51.5 | 888 | 51.2 | 991 | 53.4 | 386 | 53.5 | 991 | 52.2 | 1071 | 52.4 |
|  | |  |  |  |  |  | |  |  |  |  |  | |
| Age Group | |  |  |  |  |  |  |  |  |  |  |  |  |
| 0-4 | | 29 | 4.3 | 179 | 10.1 | 152 | 8.2 | 57 | 7.9 | 180 | 9.5 | 183 | 9.0 |
| 5-18 | | 176 | 25.9 | 451 | 25.5 | 447 | 24.1 | 206 | 28.5 | 505 | 26.6 | 529 | 25.9 |
| 19-39 | | 260 | 38.3 | 674 | 38.1 | 724 | 39.0 | 264 | 36.6 | 690 | 36.3 | 737 | 36.1 |
| 40-59 | | 168 | 24.7 | 325 | 18.4 | 380 | 20.5 | 141 | 19.5 | 354 | 18.7 | 380 | 18.6 |
| 60+ | | 46 | 6.8 | 141 | 8.0 | 154 | 8.3 | 54 | 7.5 | 169 | 8.9 | 214 | 10.5 |
|  | |  |  |  |  |  |  |  |  |  |  |  |  |
| *Pregnant women | | 11/223 | 4.9 | 20/549 | 3.6 | ^#^46/639 | 7.2 | *7/227 | 3.1 | 30/585 | 5.1 | 33/622 | 5.3 |
|  | |  |  |  |  |  |  |  |  |  |  |  |  |
| Reported diagnoses/ conditions: | |  |  |  |  |  |  |  |  |  |  |  |  |
| **Hypertension | | 90/555 | 16.2 | 186/1220 | 15.3 | 242/1357 | 17.8 | **92/548 | 16.8 | 240/1334 | 18.0 | 298/1452 | 20.5 |
| **Diabetes | | ^#^38/517 | 7.4 | 44/1220 | 3.6 | 48/1357 | 3.5 | ^#^31/503 | 6.2 | 61/1334 | 4.6 | 77/1452 | 5.3 |
| ****HIV | Overall | 90/679 | 13.3 | 122/1757 | 6.9 | 182/1834 | 9.9 | 55/722 | 7.6 | 149/1862 | 8.0 | 182/2015 | 9.0 |
|  | 15-49 years | 71/405 | 17.5 | 96/994 | 9.7 | 146/1053 | 13.9 | 43/414 | 10.4 | 111/1026 | 10.8 | 135/1082 | 12.5 |
| Persistent cough | | ^#^3/673 | 0.5 | 17/1761 | 1.0 | 19/1843 | 1.0 | ^#^12/713 | 1.7 | 32/1886 | 1.7 | 32/2028 | 1.6 |
| Requiring Care | |  |  |  |  |  |  |  |  |  |  |  |  |
| ^#^HBC | | ^#^4/639 | 0.6 | 10/1560 | 0.6 | 8/1671 | 0.5 | ^#^3/656 | 0.5 | 9/1685 | 0.5 | 11/1808 | 0.6 |
| Wound care | | 2/679 | 0.3 | 6/1770 | 0.3 | 5 | 0.3 | 3/722 | 0.4 | 5/1898 | 0.3 | 11 | 0.5 |
|  | |  |  |  |  |  |  |  |  |  |  |  |  |
| ^a^Households where a member reported a health condition | | 127/209 | 60.8 | 314 | 62.7 | 410 | 66.0 | 142/206 | 68.9 | 339 | 67.3 | 412 | 66.3 |
|  | |  |  |  |  |  |  |  |  |  |  |  |  |
| Number of health care needs reported | |  |  |  |  |  |  |  |  |  |  |  |  |
| 0 | | 82 | 39.2 | 187 | 37.3 | 211 | 34.0 | 64 | 31.1 | 165 | 32.7 | 209/621 | 33.7 |
| 1 | | 67 | 32.1 | 195 | 38.9 | 219 | 35.3 | 77 | 37.4 | 172 | 34.1 | 191/621 | 30.8 |
| 2 | | 37 | 17.7 | 83 | 16.6 | 123 | 19.8 | 46 | 22.3 | 106 | 21.0 | 112 | 18.0 |
| 3 | | 18 | 8.6 | 24 | 4.8 | 45 | 7.2 | 16 | 7.8 | 43 | 8.5 | 63 | 10.1 |
| 4 or more | | 5 | 2.4 | 12 | 2.4 | 23 | 3.7 | 3 | 1.5 | 18 | 3.6 | 46 | 7.4 |

*Proportion out of total number of women in the reproductive age group – 12-49 years (women who reported pregnancy unknown were excluded from denominator/numerator)

^**^ Denominator is everyone above 15 years according to the 2017 national prevalence for diabetes in South Africa (<http://www.kznhealth.gov.za/family/SEMDS-2017-Guidelines.pdf>) for hypertension, <http://www.health.gov.za/index.php/component/phocadownload/category/539-sadhs-south-africa-demographic-and-health-survey-report?download=3325:sadhs-2016-report>

****Excluded: those who said they don’t know their HIV status

^#^Has missing data/ as percentage of those older than 5 years

^a^Health conditions (any household member <5 years or has hypertension or diabetes or HIV or TB or cough or Home-based care or wound or pregnant)

**Supplementary Table 3 Factors associated with being visited (unadjusted and adjusted regression analysis): both sites combined**

| **S/N** | **Variable** | | **Unadjusted** | | **Adjusted (** without interaction**)** | | |
| --- | --- | --- | --- | --- | --- | --- | --- |
|  |  |  | **OR** | **95% CI** | **OR** | **95% CI** | **P value** |
| 1 | Site | Usi (Ref) |  |  | **Ref** | | |
|  |  | Vis | 1.10 | 0.90-1.34 | 1.14 | 0.89-1.46 | 0.291 |
| 2 | Dwelling type | House (Ref) |  |  | **Ref** | | |
|  |  | Informal | 1.10 | 0.91-1.35 | 1.20 | 0.93-1.55 | 0.152 |
| 3 | Under 5 | No (Ref) |  |  | **Ref** | | |
|  |  | Yes | 1.32 | 1.06-1.65 | **1.35** | **1.05-1.73** | **0.017** |
| 4 | Elderly (≥60 years) | No (Ref) |  |  | **Ref** | | |
|  |  | Yes | 2.31 | 1.87-2.85 | **2.09** | **1.61-2.71** | **0.000** |
| 5 | Hypertension | No (Ref) |  |  | **Ref** | | |
|  |  | Yes | 1.87 | 1.53-2.28 | **1.59** | **1.25-2.02** | **0.000** |
| 7 | HIV | No (Ref) |  |  | **Ref** | | |
|  |  | Yes | 1.55 | 1.24-1.94 | **1.43** | **1.00-2.06** | **0.049** |
| 8 | TB | No (Ref) |  |  | **Ref** | | |
|  |  | Yes | 2.13 | 1.22-3.72 | **2.28** | **1.23-4.23** | **0.009** |
| 13 | Time | 1 (baseline) (ref) |  |  | **ref** | | |
|  |  | 2 (endline) | 2.73 | 1.89-3.94 | **2.65** | **1.78-3.95** | **0.000** |
|  |  | 3 (post-endline) | 2.35 | 1.64-3.38 | **2.39** | **1.61-3.53** | **0.000** |
